# Incorporating Pathomics Into IgAN Risk Prediction

**DOI:** 10.1016/j.ekir.2026.106708

**Published:** 2026-07-22

**Authors:** David L. Hölscher, Sean J. Barbour, Rosanna Coppo, Lee Er, Roman D. Bülow, Martin Strauch, Vladimir Tesar, Jonathan Barratt, Ian S.D. Roberts, Peter Boor, International IgA Nephropathy Network, M.L. Russo, S. Troyanov, H.T. Cook, I.S.D. Roberts, V. Tesar, D. Maixnerova, S. Lundberg, L. Gesualdo, F. Emma, F. Diomedi, G. Beltrame, C. Rollino, A. Amore, R. Camilla, L. Peruzzi, M. Praga, S. Feriozzi, R. Polci, G. Segoloni, L. Colla, A. Pani, D. Piras, A. Angioi, G. Cancarini, S. Ravera, M. Durlik, E. Moggia, J. Ballarin, S. Di Giulio, F. Pugliese, I. Serriello, Y. Caliskan, M. Sever, I. Kilicaslan, F. Locatelli, L. Del Vecchio, J.F.M. Wetzels, H. Peters, U. Berg, F. Carvalho, A.C. da Costa Ferreira, M. Maggio, A. Wiecek, M. Ots-Rosenberg, R. Magistroni, R. Topaloglu, Y. Bilginer, M. D’Amico, M. Stangou, F. Giacchino, D. Goumenos, P. Kalliakmani, M. Papasotiriou, K. Galesic, C. Geddes, K. Siamopoulos, O. Balafa, M. Galliani, P. Stratta, M. Quaglia, R. Bergia, R. Cravero, M. Salvadori, L. Cirami, B. Fellstrom, H. Kloster Smerud, F. Ferrario, T. Stellato, J. Egido, C. Martin, J. Floege, F. Eitner, A. Lupo, P. Bernich, P. Menè, M. Morosetti, C. van Kooten, T. Rabelink, M.E.J. Reinders, J.M. Boria Grinyo, S. Cusinato, L. Benozzi, S. Savoldi, C. Licata, M. Mizerska-Wasiak, G. Martina, A. Messuerotti, A. Dal Canton, C. Esposito, C. Migotto, G. Triolo, F. Mariano, C. Pozzi, R. Boero, S. Bellur, G. Mazzucco, C. Giannakakis, E. Honsova, B. Sundelin, A.M. Di Palma, F. Ferrario, E. Gutiérrez, A.M. Asunis, J. Barratt, R. Tardanico, A. Perkowska-Ptasinska, J. Arce Terroba, M. Fortunato, A. Pantzaki, Y. Ozluk, E. Steenbergen, M. Soderberg, Z. Riispere, L. Furci, D. Orhan, D. Kipgen, D. Casartelli, D. Galesic Ljubanovic, H. Gakiopoulou, E. Bertoni, P. Cannata Ortiz, H. Karkoszka, H.J. Groene, A. Stoppacciaro, I. Bajema, J. Bruijn, X. Fulladosa Oliveras, J. Maldyk, E. Ioachim, N. Bavbek, T. Cook, S. Troyanov, C. Alpers, A. Amore, J. Barratt, F. Berthoux, S. Bonsib, J. Bruijn, V. D’Agati, G. D’Amico, S. Emancipator, F. Emmal, F. Ferrario, F. Fervenza, S. Florquin, A. Fogo, C. Geddes, H. Groene, M. Haas, P. Hill, R. Hogg, S. Hsu, T. Hunley, Hladunewich, C. Jennette, K. Joh, B. Julian, T. Kawamura, F. Lai, C. Leung, L. Li, P. Li, Z. Liu, A. Massat, B. Mackinnon, S. Mezzano, F. Schena, Y. Tomino, P. Walker, H. Wang, J. Weening, N. Yoshikawa, Cai-Hong Zeng, Sufang Shi, C. Nogi, H. Suzuki, K. Koike, K. Hirano, T. Kawamura, T. Yokoo, M. Hanai, K. Fukami, K. Takahashi, Y. Yuzawa, M. Niwa, Y. Yasuda, S. Maruyama, D. Ichikawa, T. Suzuki, S. Shirai, A. Fukuda, S. Fujimoto, H. Trimarchi

**Affiliations:** 1 Institute of Pathology, RWTH Aachen University Hospital, Aachen, Germany; 2 Department of Nephrology and Immunology, RWTH Aachen University Hospital, Aachen, Germany; 3 Division of Nephrology, University of British Columbia, Vancouver, Canada; 4 BC Renal, Vancouver, Canada; 5 Fondazione Ricerca Molinette, Torino, Italy; 6 Regina Margherita Children’s University Hospital, Torino, Italy; 7 Department of Nephrology, 1^st^ Faculty of Medicine and General University Hospital, Charles University, Prague, Czech Republic; 8 John Walls Renal Unit, University Hospital of Leicester National Health Service Trust, Leicester, UK; 9 Department of Cardiovascular Sciences, University of Leicester, Leicester, UK; 10 Department of Cellular Pathology, Oxford University Hospitals National Health Services Foundation Trust, Oxford, UK

**Keywords:** deep learning, IgA nephropathy, pathomics, risk prediction

## Abstract

**Introduction:**

IgA nephropathy (IgAN) is the most common glomerulonephritis worldwide, with heterogeneous progression rates. The International IgAN Prediction Tool (IIgAN-PT) enables individualized risk prediction using clinical characteristics and histopathological scoring. However, traditional histopathological scoring is semiquantitative and prone to interobserver variability. Deep learning-based, automated quantification of digitized histopathology enables the extraction of quantitative morphological biomarkers (pathomics). Here, we evaluated the added prognostic value of pathomics over traditional histopathology and its potential integration into the IIgAN-PT.

**Methods:**

We analyzed a multicenter European cohort of 555 adults with biopsy-proven IgAN (VALIGA), including centralized histopathology assessment. We assessed the potential of 25 clinically interpretable candidate pathomics predictors across 2 clinically relevant settings by (i) comparing pathomics with traditional histopathology (MEST-C [mesangial hypercellularity (M), endocapillary hypercellularity (E), segmental glomerulosclerosis (S), tubular atrophy and/or interstitial fibrosis (T) and crescents (C)]) and (ii) extending the IIgAN-PT with pathomics. Model performance was evaluated by overall fit, discrimination, reclassification, and calibration.

**Results:**

In a direct comparison, pathomics slightly outperformed the MEST-C score when used alone (Δ C-statistic 0.04 (95% confidence interval [CI]: 0.03–0.06)) but was marginally worse after adjusting for clinical predictors (Δ C-statistic -0.034 [95% CI: −0.042 to −0.027]). Patient stratification into clinical risk groups was consistent between models. Combining pathomics and traditional histopathology led to minor improvements over the current IIgAN-PT (without ethnicity, Δ C-statistic 0.024 [95% CI: 0.019–0.028]). Pathomics variables captured chronic morphological changes including glomerular deformation, tubular shrinkage, and arterial luminal narrowing.

**Conclusion:**

Our findings indicate that pathomics-derived digital tissue biomarkers provide an additional layer of information and are noninferior to MEST-C, supporting more standardized, robust, and reproducible risk stratification in IgAN.

## Graphical abstract

**Figure:**
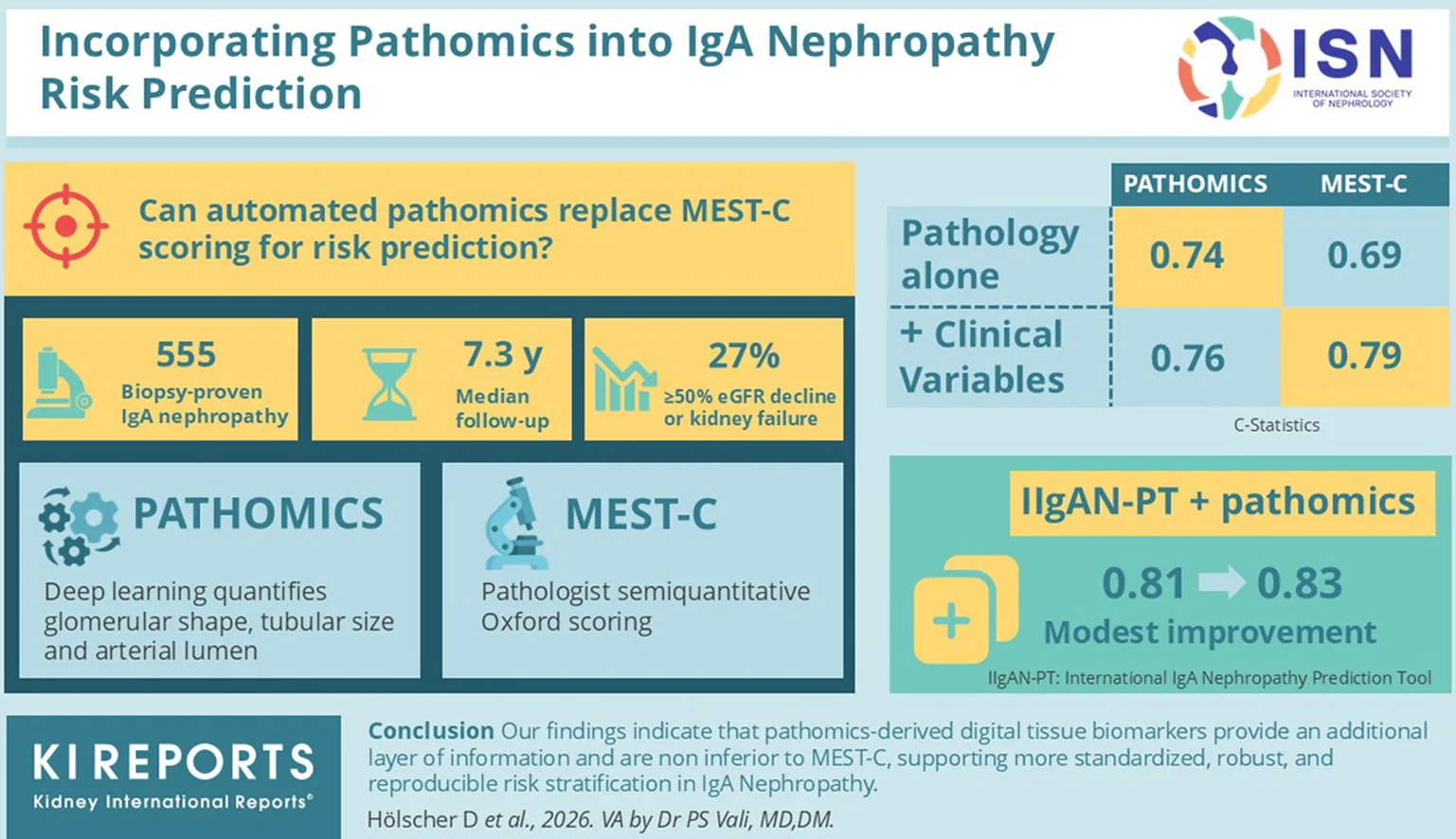

IgAN is the most prevalent glomerulonephritis worldwide and a leading cause of kidney failure in adults and children.^1^^,^^2^ IgAN remains a clinically heterogeneous entity, with presentations ranging from asymptomatic microscopic hematuria to progressive kidney failure.^3^ Similar to its initial presentation, the trajectory of disease is also highly variable, with less than 10% to over 50% of patients progressing to kidney failure within 10 to 20 years after diagnosis, depending on regional variability and further risk factors.^4^^,^^5^ To improve the estimation of risk for disease progression at the time of kidney biopsy, the IIgAN-PT was developed and validated in different patient populations worldwide.^6^^,^^7^ The IIgAN-PT has been further updated to be applicable for children^8^ and is the recommended method of risk stratification for IgAN according to the international Kidney Disease: Improving Global Outcomes glomerulonephritis guidelines.^9^ The adult IIgAN-PT comprises 2 validated Cox survival models that incorporate clinical risk factors such as baseline estimated glomerular filtration rate (eGFR) and proteinuria, and pathologists-derived histopathological scores of the Oxford MEST-C classification. The presence of crescents, which was later incorporated into the Oxford classification,^10^ was not included in the IIgAN-PT because it was highly correlated with race and/or ethnicity and confounded by subsequent use of immunosuppression.^6^ Each of the MEST scores was previously validated as a predictor of disease progression.^11^, ^12^, ^13^ However, these semiquantitative (categorical) scores are often subject to considerable interobserver variability.^14^

Recent innovations in computational nephropathology, particularly the introduction of pathomics,^15^, ^16^, ^17^ could address these limitations by enabling the automated extraction of quantitative, reproducible, and interpretable biomarkers directly from digitized kidney biopsy slides. These deep learning-based digital biomarkers have the potential to further standardize and enrich the histological assessment of IgAN. We previously showed that pathomics features are independent predictors of long-term disease progression in IgAN.^15^

Here, we assessed the prognostic value of pathomics-derived features in predicting IgAN progression within the IIgAN-PT using the well-characterized multicenter European Validation Study of the Oxford Classification of IgAN (VALIGA) cohort.^11^^,^^18^ We explore the integration of pathomics-based digital biomarkers into the established IIgAN-PT and directly compare their predictive performance to conventional histopathological scoring, providing a blueprint for the integration of quantitative computational pathology and pathomics into clinical disease prediction.

## Methods

### Study Population

The study population comprised the VALIGA cohort of patients that was used in the derivation of the IIgAN-PT, as described previously.^6^^,^^11^ This cohort includes patients with biopsy-proven IgAN, available MEST-C scores, age ≥ 18 years, who did not have kidney failure at the time of biopsy, and who had eGFR available at biopsy and at least once during follow-up. We additionally excluded patients without available pathomics data as not all cases from the original cohort could be identified and digitized (scanned). Additionally, cases were excluded because of broken glass slides, missing slide labels, insufficient staining, or large artifacts, as described previously.^15^

### Variable Definitions

Definitions of predictor variables were the same as those used in the IIgAN-PT analysis for the models to predict risk at the time of biopsy. Mean arterial blood pressure, eGFR (using the creatinine-based Chronic Kidney Disease Epidemiology Collaboration 2009 formula), and proteinuria were the closest values within 6 months of kidney biopsy.^6^^,^^19^ Proteinuria was taken from 24-hour urine collections, when these were not available, daily excretion of urinary protein was estimated from spot urine protein to creatinine ratios.^20^ The Oxford MEST-C scores were based on established criteria, except that crescents were considered as the presence or absence of cellular or fibrocellular crescents.^10^^,^^12^^,^^21^ The percentage of glomeruli with crescents was not available. Ethnicity was self-reported as either White, Chinese, Japanese, or other. Age and previous use of immunosuppression or medications that inhibit the renin-angiotensin system were determined at the time of kidney biopsy. The primary outcome was a composite of the first occurrence of kidney failure (eGFR < 15 ml/min per 1.73 m^2^, dialysis, or kidney transplantation) or a permanent reduction in eGFR to below 50% of the value at biopsy.

### Pathomics Variables

Glass slides of all kidney biopsies from the VALIGA cohort were digitized locally at the Institute of Pathology, RWTH Aachen, using an Aperio AT2 whole-slide scanner with a 40x objective (Leica Biosystems, Wetzlar, Germany). A total of 648 Periodic Acid Schiff -stained whole-slide images were analyzed using our previously published pathomics framework.^15^ This framework incorporates deep learning-based segmentation of the following 6 relevant kidney tissue compartments: glomeruli, glomerular tufts, tubules, arteries, their respective lumens, and nontissue background including large veins; followed by the automated extraction of morphometric features such as area, diameter, distance between segmented instances, or shape descriptors. In total, 25 pathomics predictors were extracted (Supplementary Table S1). Pathomics features of segmented structures were aggregated on a patient-level using the median of the respective feature distribution for compatibility with tabular data.

### Statistical Analysis

#### Predictor Selection

The IIgAN-PT at biopsy comprises 2 Cox proportional hazards models of time from biopsy to the primary outcome, right-censored at death or the end of follow-up, with or without ethnicity as a predictor in addition to age, eGFR, mean arterial blood pressure, proteinuria at biopsy, previous use of renin-angiotensin system inhibitors and immunosuppression; and the MEST histology scores.^6^ The analysis strategy was designed to determine if the pathomics variables could replace the Oxford classification or if they could be added to the IIgAN-PT for improved individual patient risk stratification. Accordingly, comparison of pathomics variables with the Oxford classification was based on the complete MEST-C score, whereas integration into the IIgAN-PT considered only the MEST components, given that the C component is not included in the IIgAN-PT. Because some of the 25 pathomics variables might reflect similar histology characteristics and are highly correlated, the first step was to use a clustering analysis using PROC VARCLUS (SAS Institute, Cary, NC) to reduce the number of candidate predictors by identifying disjoint clusters of pathomics variables. This resulted in 10 pathomics variables considered in subsequent analyses. For each variable, the proportional hazards and linearity assumptions were assessed using an interaction with log (time) and using restricted cubic splines, respectively.

#### Comparing Pathomics to the Oxford Classification

To determine if the pathomics variables could replace the Oxford classification when considered alone and in addition to clinical variables for individual patient risk stratification, they were included in 2 new models, 1 that also contained the same mandatory clinical predictors that are in the IIgAN-PT (age, eGFR, mean arterial blood pressure and proteinuria at biopsy; and previous use of renin-angiotensin system inhibitors and immunosuppression). The same transformations were used for eGFR (square root) and proteinuria (log) as in the IIgAN-PT to maintain a linear functional form. Ethnicity was not included because the VALIGA cohort is mostly White. Beta-coefficients were estimated using Cox proportional hazard regression (for models with MEST-C or pathomics alone) and ridge regression (for models with MEST-C or pathomics including clinical predictors) to penalize estimates for overfitting while ensuring the clinical predictors were forced into the model.^22^^,^^23^ The prediction performance of these new models was compared with alternative models that contained the MEST-C scores instead of the pathomics variables. For the models including clinical predictors, it would not have been appropriate to instead compare the prediction performance of this new model to the original IIgAN-PT because the former was derived in the same cohort in which it was being evaluated whereas the latter was derived in a different cohort. By using 2 models that were both fit in the same dataset, any differences in prediction performance could be attributable to the choice of pathology variables. To assess the models’ ability to differentiate between clinically meaningful risk groups, we stratified patients based on the 16th, 50th, and 84th percentiles of the linear predictor^24^ and plotted Kaplan-Meier curves for the resulting 4 risk groups for both pathomics and MEST-C models including clinical predictors.

#### Incorporating Pathomics Into the IIgAN-PT

To determine if the pathomics variables improve risk prediction if they are added to the IIgAN-PT, we used the analysis strategy proposed by Steyerberg in which an updated version of the tool was generated that incorporated the pathomics variables by including them in a model with the linear predictor from the IIgAN-PT as an offset variable.^25^ The linear predictor for each patient was generated using the predictor values in the analytic dataset and the beta-coefficients from the IIgAN-PT. Because of the number of outcome events in the cohort, this was done using least absolute shrinkage and selection operator regression which shrinks beta-coefficients to protect against overfitting and assigns coefficients a value of zero for unimportant predictors.^26^ The predictive performance of the updated tool that incorporated pathomics variables was compared with the original IIgAN-PT.

#### Model Performance

The prediction performance of the various models was assessed using model fit, determined by R^2^_D_ and the Akaike Information Criterion, discrimination as measured by the C-statistic adapted for censoring^24^^,^^27^ and the integrated discrimination index,^28^ and reclassification as measured by the continuous reclassification index.^29^ Confidence intervals were generated using 100 bootstrap samples. Calibration for a specific time horizon was evaluated using smoothed calibration plots of predicted versus observed risk, and using the integrated calibration index, which is a weighted difference between predicted and observed risk that quantifies the amount of miscalibration.^30^ Internal validation was performed using bootstrap resampling to generate optimism-corrected measures of prediction performance.^25^

Missing data for predictor variables in the IIgAN-PT were very infrequent (0–7.7%) and were imputed using multiple imputation with chained equations.^31^, ^32^, ^33^ Descriptive variables were presented as median (interquartile range[IQR]), or count (frequency). All analyses were performed using SAS Version 9.4 (SAS Institute, Cary, NC) and figures were generated using RStudio (version 2025.05.0+496; Posit Software, PBC, Boston, MA). This project was approved by the University of British Columbia research ethics board, with waived patient consent. Results were presented according to the STROBE guidelines (Supplementary Table S2).

## Results

**Figure 1:**
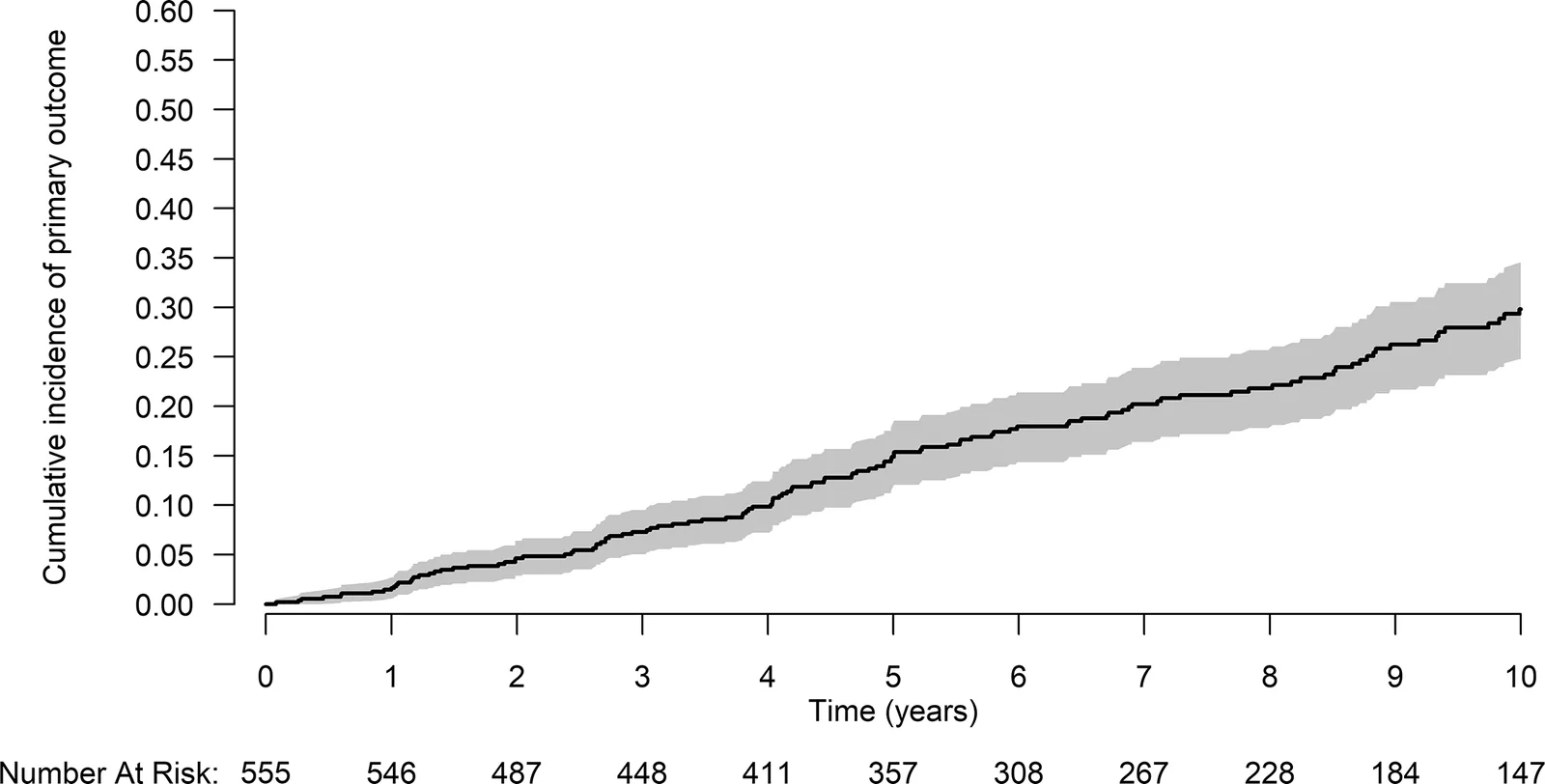
The cumulative incidence of the primary outcome (50% decline in eGFR or kidney failure). The shaded regions indicate 95% confidence intervals. eGFR, estimated glomerular filtration rate.

The analytic cohort comprised 555 patients and is described in Table 1, Supplementary Table S3 and Supplementary Figure S1. The median age was 37.9 years (IQR: 28.3–48.6), 72% were male and 97% were White. At the time of biopsy, median proteinuria was 1.3 g/day (IQR: 0.7–2.6) and eGFR was 72 ml/min per 1.73m^2^ (IQR: 46–95). Over a median follow-up duration of 7.3 years (IQR: 4.2–11.1), 509 patients were treated with renin-angiotensin system inhibitors (92%), and 217 were treated with immunosuppression (39%). The primary outcome (50% decrease in eGFR or kidney failure) occurred in 148 patients (27%) with a 7- and 10-year risk of 20.2% (95%CI: 16.4–23.9) and 29.8% (95% CI: 24.8–34.5), respectively (Figure 1).

**Table 1:** Description of the analytic cohort

Characteristics	Analytic cohort
Number of patients	555
Duration of follow-up (yrs)	7.3 (4.2–11.1)
Year of biopsy	2004 (2000–2007)
Age (yrs)	37.9 (28.3–48.6)
Male sex	402 (72%)
Ethnicity	
White	540 (97%)
Other	15 (3%)
Creatinine at biopsy (μmol/l)	106 (86–148)
eGFR at biopsy (ml/min per1.73 m^2^)	72 (46–95)
MAP at biopsy (mm Hg)	98.3 (92.3–106.7)
Proteinuria at biopsy (g/d)	1.3 (0.7–2.6)
Pathology	
M1	165 (30%)
E1	59 (11%)
S1	433 (78%)
T1	121 (22%)
T2	21 (4%)
Crescents	54 (10%)
Use of RASi at biopsy	246 (44%)
Use of RASi after biopsy	509 (92%)
Immunosuppression before biopsy	69 (12%)
Immunosuppression after biopsy	217 (39%)
Primary outcome	
50% decrease in eGFR	129 (23%)
Kidney failure	127 (23%)
Composite	148 (27%)

eGFR, estimated glomerular filtration rate; MAP, mean arterial blood pressure; RASi, use of medications that inhibit the renin-angiotensin system.

Data presented as median (IQR) or count (frequency). The primary outcome was the first occurrence of either a permanent 50% decline in eGFR from that at biopsy or kidney failure.

### Identifying Pathomics Predictor Variables

There were 25 candidate pathomics variables, which are summarized in Supplementary Table S1. The correlation between pathomics variables was assessed using a clustering analysis (Supplementary Table S4 and Supplementary Figure S2). This resulted in 10 distinct clusters, and those variables from each cluster with the smallest 1-R^2^ ratio have the strongest correlation with other variables in the same cluster and the least correlation with variables in other clusters and were therefore selected for subsequent analyses. The selected pathomics variables were tubular diameter, tuft eccentricity, arterial diameter, tuft area, glomerular circularity, arterial lumen diameter, glomerular distance, arterial distance, Bowman’s area, and glomerular eccentricity. Glomerular tuft area and glomerular circularity exhibited the strongest correlation with eGFR (Spearman’s rank correlation coefficient of 0.43 and 0.48, respectively) and proteinuria (-0.17 for both) at time of kidney biopsy (Supplementary Figure S3).

### Comparing Pathomics to MEST-C for Risk Stratification

**Figure 2:**
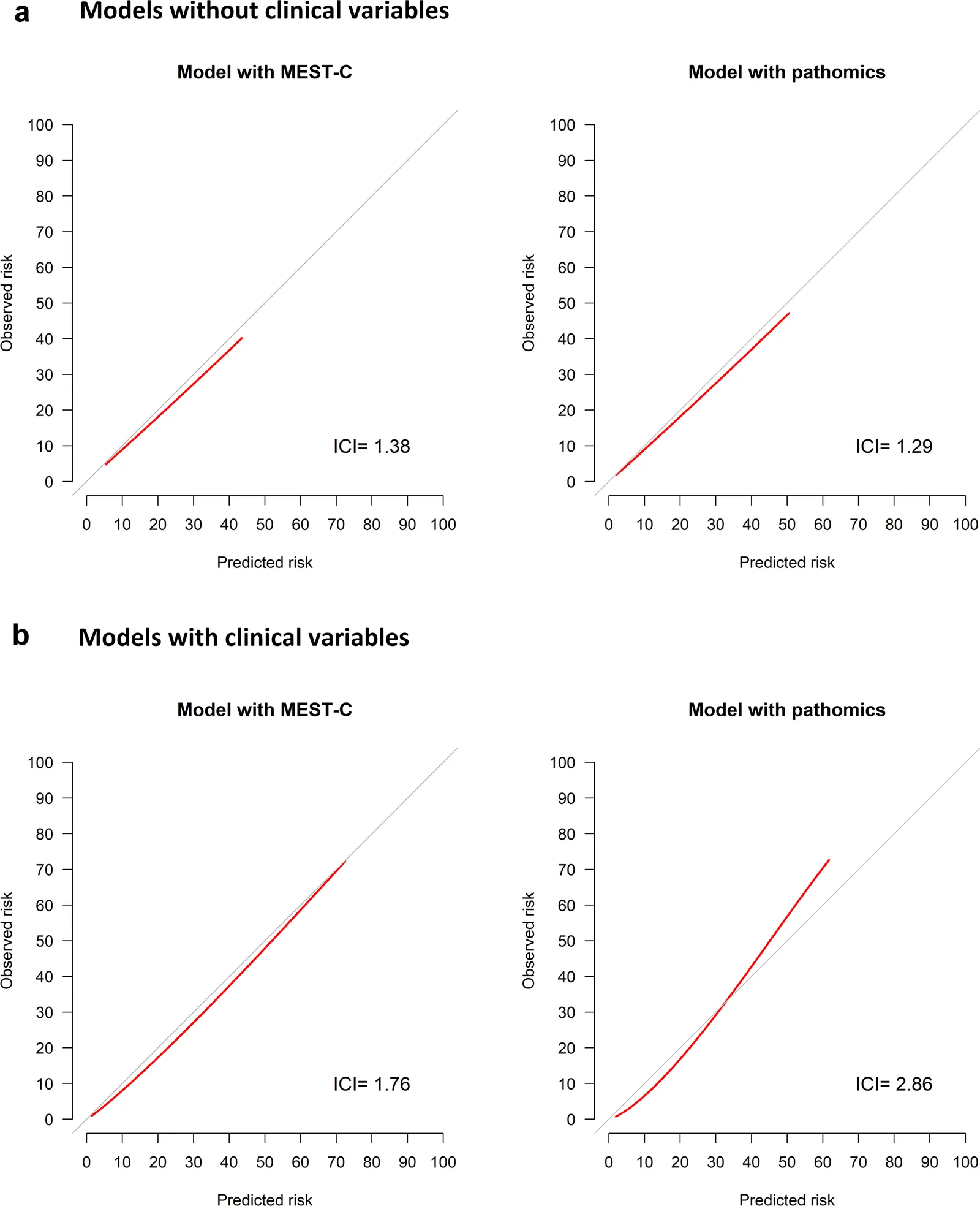
Calibration curves depicting the predicted versus observed 5-year risks of the primary outcome using the multivariable models of MEST-C and pathomics variables with or without clinical predictors. The predicted 5-year risks are from the prediction models, and observed 5-year risks are estimated using a flexible adaptive hazard regression model with the complementary log-log of the predicted 5-year risk as the covariate, as proposed by Austin *et al.*^30^ The grey line represents perfect calibration in which predicted and observed risks are identical. ICI, integrated calibration index; MEST-C, mesangial hypercellularity (M), endocapillary hypercellularity (E), segmental glomerulosclerosis (S), tubular atrophy and/or interstitial fibrosis (T) ,and crescents (C).

**Figure 3:**
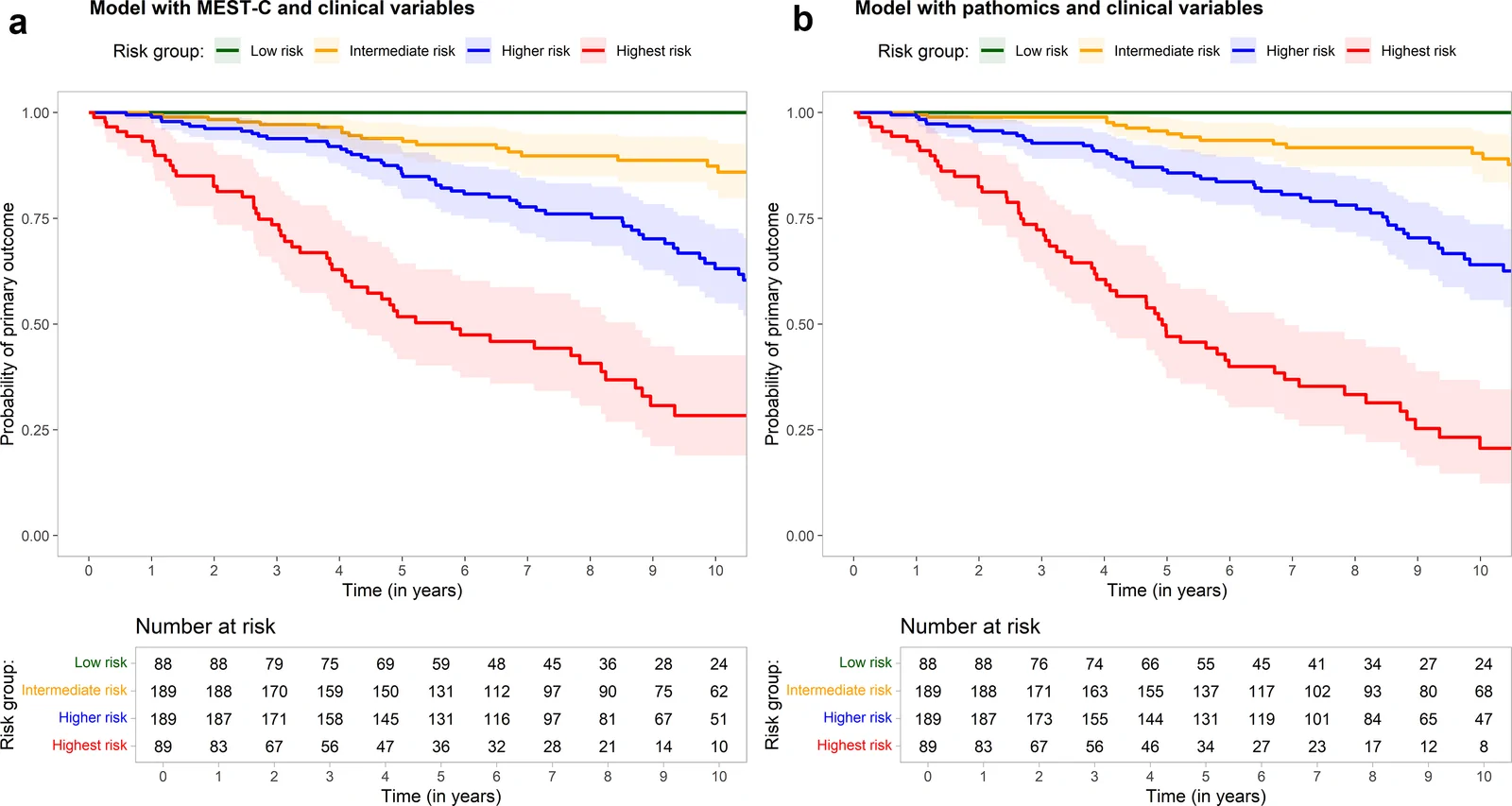
Kaplan-Meier survival curves with numbers at risk for 4 risk groups stratified by percentiles of the linear predictors derived from (a) the MEST-C model and (b) the pathomics model, both adjusted for clinical variables. Shaded areas represent 95% confidence intervals. MEST-C, mesangial hypercellularity (M), endocapillary hypercellularity (E), segmental glomerulosclerosis (S), tubular atrophy and/or interstitial fibrosis (T) ,and crescents (C).

To determine if the pathomics variables can replace the MEST-C scores for patient risk stratification at the time of biopsy, multivariable models were fit with and without clinical predictors in addition to either the 10 pathomics variables or the MEST-C scores. Beta-coefficients were estimated using ridge regression to penalize estimates for overfitting (Table 2). Considering no additional clinical variables, the model with pathomics had a better goodness of fit, that is, a lower Akaike Information Criterion and comparable R^2^_D_, better discrimination given an increased C-statistic, and no significant difference in integrated discrimination index, continuous reclassification index, and integrated calibration index (Table 3, Figure 2). When accounting for the clinical predictors included in the IIgAN-PT, the model with MEST-C had a better Akaike Information Criterion, improved discrimination, and no significant difference in reclassification and calibration (Table 3). The results were similar after internal validation (Supplementary Table S5), although generally the model with pathomics had a lower R^2^_D_ compared with the model with MEST-C. Overall, these results suggest slightly improved risk prediction when using pathomics instead of MEST-C without clinical variables, but marginally better performance of MEST-C compared with pathomics when considering the additional clinical variables included in the IIgAN-PT. Stratification of patients into 4 clinical risk groups yielded similar survival curves between the pathomics and MEST-C models including clinical variables (Figure 3).

**Table 2:** Results of the ridge regression multivariable models with either MEST-C or pathomics variables

Characteristics	MEST-C model		Pathomics model	
	β-coefficient	Hazard ratio	β-coefficient	Hazard ratio
Age (yrs)	−0.0015	1.00	0.0017	1.00
eGFR (ml/min per 1.73 m^2^)^a^	−0.2084	0.81	−0.1590	0.85
MAP (mm Hg)	0.0169	1.02	0.0153	1.02
Proteinuria (g/day)^b^	0.4569	1.58	0.3176	1.37
Previous RASi	0.3132	1.37	0.2288	1.26
Previous immunosuppression	0.1507	1.16	0.2169	1.24
M1	0.1166	1.12		
E1	0.1229	1.13		
S1	0.1761	1.19		
T1	0.7937	2.21		
T2	0.4843	1.62		
Crescents	-0.1657	0.85		
Tubular diameter (μm)^c^			−0.0295	0.97
Tuft eccentricity^c^			0.8941	2.45
Arterial diameter (μm)^c^			0.0102	1.01
Tuft area (μm^2^)^c^ (per 1.000)			−0.0360	0.96
Glomerular circularity^c^			−0.9503	0.39
Arterial lumen diameter (μm)^c^			−0.0458	0.96
Glomerular distance (per 1.000μm)^c^			1.6299	5.10
Arterial distance (μm)^c^			−0.0100	0.99
Bowman area (per 1.000μm^2^)^c^			0.0336	1.03
Glomerular eccentricity^c^			−0.8808	0.41

eGFR, estimated glomerular filtration rate; MAP, mean arterial blood pressure; RASi, use of medications that inhibit the renin-angiotensin system.

Ridge regression will shrink beta-coefficients to reduce overfitting and does not provide confidence intervals.

a eGFR was square root transformed, as such the hazard ratio is per 1 square unit increase.

b Proteinuria was log transformed, as such the hazard ratio is per 1 log unit increase.

c Hazard ratios are provided per 1 unit increase in the pathomics variables, unless otherwise specified.

**Table 3:** Measures of prediction performance for the multivariable models of MEST-C and pathomics variables with or without clinical predictors

Aspect	Measure of performance	Model with MEST-C	Model with pathomics
Models without clinical variables			
Goodness of fit	AIC	1546	1539
	R^2^_D_ (95% CI)	12.9% (8.6%–17.8%)	13.0% (8.6%– 17.9%)
Discrimination, reclassification	C-statistic (95% CI)	0.69 (0.68–0.70)	0.74 (0.73–0.75)
	Δ C-statistic (95%CI)	Reference	0.04 (0.03–0.06)
	Event cNRI (95% CI)	Reference	0.027 (−0.446 to 0.543)
	Nonevent cNRI (95% CI)	Reference	−0.116 (−0.377 to 0.763)
	IDI (95% CI)	Reference	0.002 (−0.072 to 0.049)
Calibration	ICI (95% CI)	1.38 (0.05–2.27)	1.29 (0.05–2.83)
	Δ ICI (95% CI)	Reference	−0.09 (−1.50 to 2.34)
Models with clinical variables			
Overall performance	AIC	1488	1501
	R^2^_D_ (95% CI)	27.0% (20.8%–34.3%)	27.6% (21.8%–33.7%)
Discrimination, reclassification	C-statistic (95% CI)	0.79 (0.78–0.81)	0.76 (0.75–0.77)
	Δ C-statistic (95% CI)	Reference	−0.034 (−0.042 to −0.027)
	Event cNRI (95% CI)	Reference	−0.188 (−0.464 to 0.561)
	Nonevent cNRI (95% CI)	Reference	−0.319 (−0.379 to 0.769)
	IDI (95% CI)	Reference	−0.057 (−0.072 to 0.052)
Calibration	ICI (95% CI)	1.76 (0.73–11.56)	2.86 (0.86–5.68)
	Δ ICI (95% CI)	Reference	1.10 (-9.28 to 3.44)

MAP, mean arterial blood pressure; RASi, use of medications that inhibit the renin-angiotensin system.

AIC (Akaike Information Criterion) and R^2^_D_ are measures of model fit, with lower and higher values respectively indicating better model fit. The C-statistic was evaluated at a 5-year time horizon. The continuous NRI at the 5-year time horizon is the net proportion of events reclassified correctly (event cNRI), and the net proportion of nonevents reclassified correctly (nonevent cNRI) by a new model from the reference model. The IDI (integrated discrimination improvement) measures the extent to which a new model increases the average 5-year risks in events and decreases the average 5-year risk in nonevents from the reference model, with higher values indicating improvement in discrimination. The ICI (integrated calibration index) is a measure of miscalibration, with higher values indicating worse calibration and was evaluated at a 5-year time horizon. The clinical variables included in the models were age, eGFR, MAP and proteinuria at biopsy, and previous use of RASi and immunosuppression.

### Adding Pathomics Variables to the IIgAN-PT

**Figure 4:**
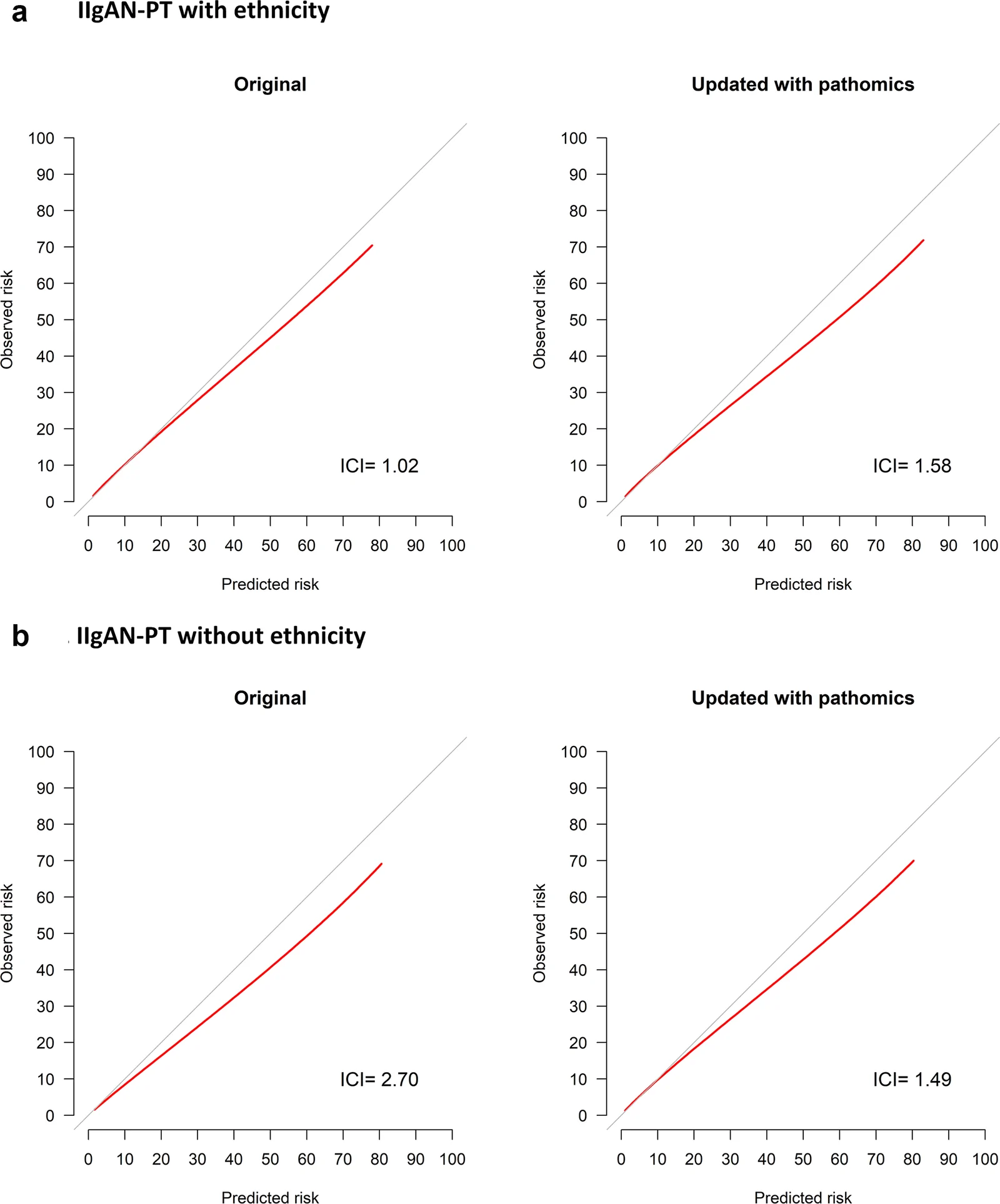
Calibration curves depicting the predicted versus observed 5-year risks of the primary outcome using the original IIgAN-PT and the updated IIgAN-PT that includes pathomics variables. The predicted 5-year risks are from the prediction models, and observed 5-year risks are estimated using a flexible adaptive hazard regression model with the complementary log-log of the predicted 5-year risk as the covariate, as proposed by Austin *et al.*^30^ The grey line represents perfect calibration in which predicted and observed risks are identical. IIgAN-PT, International IgAN Prediction Tool.

The 10 pathomics variables were added to the IIgAN-PT models at biopsy with and without ethnicity using least absolute shrinkage and selection operator regression (Table 4). For both the IIgAN-PT with and without ethnicity, decreased tubular diameter, tuft area, glomerular circularity, glomerular eccentricity, arterial distance, and arterial lumen diameter, as well as increased glomerular distance were associated with an increased risk of the primary outcome. For the IIgAN-PT without ethnicity (but not for the model with ethnicity), decreased tuft eccentricity, as well as increased arterial diameter and Bowman’s area were associated with a higher risk of the primary outcome. The prediction performances of the updated IIgAN-PT models that include the pathomics variables were compared with the performance of the original IIgAN-PT (Table 5). For both the models with and without ethnicity, we observed a slight increase in R^2^_D_ and an improved C-statistic and reclassification, particularly in the nonevent continuous reclassification index. Calibration plots are shown in Figure 4. For the model with ethnicity, calibration was worse with the addition of the pathomics variables, given by a higher integrated calibration index (1.58 vs. 1.02), whereas for the model without ethnicity, calibration was better with a lower integrated calibration index after addition of the pathomics variables (1.49 vs. 2.70). Overall, these findings suggest that adding the selected pathomics variables to the IIgAN-PT could marginally improve risk prediction at the time of biopsy.

**Table 4:** Results of the LASSO regression models in which the pathomics variables were added to the IIgAN-PT with and without ethnicity

Pathomics variables	IIgAN-PT model with ethnicity		IIgAN-PT model without ethnicity	
	β-coefficient	Hazard ratio	β-coefficient	Hazard ratio
Tubular diameter (μm)	−0.0155	0.98	−0.0178	0.98
Tuft eccentricity	0	n/a	−0.3546	0.70
Arterial diameter (μm)	0	n/a	0.0055	1.01
Tuft area (per 1000 μm^2^)	−0.0064	0.99	−0.0140	0.99
Glomerular circularity	−0.3283	0.72	−0.8876	0.41
Arterial lumen diameter (μm)	−0.0173	0.98	−0.0289	0.97
Glomerular distance (per 1000 μm)	1.5325	4.63	1.7415	5.71
Arterial distance (μm)	−0.0406	0.96	−0.0722	0.93
Bowman area (per 1000 μm^2^)	0	n/a	0.0123	1.01
Glomerular eccentricity	−0.0311	0.97	−0.6867	0.50

LASSO, least absolute shrinkage and selection operator.

The linear predictor from the IIgAN-PT for each patient was included as an offset variable in a multivariable model with all 10 pathomics variables. This allowed the model to evaluate the association of the pathomics variables with the primary outcome (50% decrease in eGFR or kidney failure) independent of the IIgAN-PT. LASSO will shrink beta-coefficients to reduce overfitting, and will assign coefficients a value of zero for pathomics variables that were not associated with the primary outcome independent of the IIgAN-PT. LASSO does not provide confidence intervals. Hazard ratios expressed per 1 unit increase in the pathomics variables.

N/A, not applicable because the beta-coefficient was shrunk to zero.

**Table 5:** Measures of prediction performance for the original IIgAN-PT compared with the updated IIgAN-PT that include pathomics variables

Aspect	Measure of performance	IIgAN-PT model with ethnicity		IIgAN-PT model without ethnicity	
		Original	With pathomics	Original	With pathomics
Goodness of fit	R^2^_D_ (95% CI)	25.4% (18.4%–32.0%)	26.3% (16.0%–31.8%)	26.1% (19.8%–32.5%)	26.7% (15.4%–31.3%)
Discrimination, reclassification	C-statistic (95% CI)	0.82 (0.80–0.84)	0.83 (0.82–0.85)	0.81 (0.80–0.83)	0.83 (0.82–0.85)
	Δ C-statistic (95%CI)	Reference	0.016 (0.013–0.019)	Reference	0.024 (0.019–0.028)
	Event cNRI (95% CI)	Reference	0.42 (−0.49 to 0.58)	Reference	−0.32 (−0.49 to −0.06)
	Nonevent cNRI (95% CI)	Reference	0.28 (0.21–0.77)	Reference	0.72 (0.65–0.77)
	IDI (95% CI)	Reference	0.038 (−0.010 to 0.052)	Reference	0.002 (−0.017 to 0.018)
Calibration	ICI (95% CI)	1.02 (0.58, 4.28)	1.58 (0.81–3.59)	2.70 (0.66–4.11)	1.49 (0.76–3.35)
	Δ ICI (95% CI)	Reference	0.56 (−2.10 to 1.09)	Reference	−1.21 (−2.75 to 1.00)

cNRI, continuous reclassification index.

R^2^_D_ is a measure of model fit, with higher values indicating better model fit. The C-statistic was evaluated at a 5-year time horizon. The continuous NRI at the 5-year time horizon is the net proportion of events reclassified correctly plus the net proportion of nonevents reclassified correctly by a new model from the reference model. The IDI (integrated discrimination improvement) measures the extent to which a new model increases the average 5-year risks in events and decreases the average 5-year risk in nonevents from the reference model, with higher values indicating improvement in discrimination. The ICI (integrated calibration index) is a measure of miscalibration, with higher values indicating worse calibration and was evaluated at a 5-year time horizon.

## Discussion

In this proof-of-concept study leveraging the retrospective, multicentric VALIGA cohort, we demonstrate that quantitative pathomics features derived from routine kidney biopsy images can be integrated into established risk prediction frameworks, such as the IIgAN-PT. Our data show that pathomics-based risk prediction could slightly improve risk prediction in comparison to the established prediction tool. Notably, several key morphometric changes, such as glomerular deformation and shrinkage of the tuft, which we previously have morphologically associated with progression towards global glomerulosclerosis, tubular shrinkage signaling atrophy, and reduction in arterial lumen reflecting intimal thickening were all associated with an increased risk of the composite outcome, providing biological plausibility for the relationships observed.^15^

There are several benefits to focusing on IgAN when studying pathomics features—it is a highly heterogenous disease with varying disease progression rates, it has an existing standardized histopathological scoring system, and a validated risk prediction model. Since the diagnosis of IgAN requires a kidney biopsy, digital image analysis and pathomics from the histological slides can be integrated in baseline assessment. Finally, the VALIGA study offered a unique multicenter dataset with centralized scoring of the MEST-C Oxford classification,^11^ allowing for comparisons of pathomics features with expert-level histopathological assessment.

Our findings demonstrate the potential clinical value of implementing computational nephropathology in routine care. Pathomics predictors led to minor improvements in overall predictive performance compared with the established prediction tool and showed marginally better predictive performance compared with MEST-C when used alone. However, the model using pathomics variables was slightly inferior to the model using MEST-C when including clinical variables. This might be because of the pathomics features mostly reflecting chronic changes, for example, glomerulosclerosis or interstitial fibrosis and tubular atrophy, which are also reflected by changes in kidney function or proteinuria. Additionally, because of sample size considerations we did not introduce interaction terms between pathomics and clinical variables, although this warrants further investigation in larger cohorts. Importantly, we observed only minor differences in model performance when comparing pathomics to MEST-C and when integrating pathomics into the IIgAN-PT. These small differences in discrimination and calibration between MEST-C and pathomics did not translate into meaningful changes in clinical utility when stratifying patients into risk groups, suggesting overall noninferiority of pathomics to MEST-C for risk prediction in the VALIGA cohort.

In clinical practice, the main advantage of pathomics variables would not be the incremental gain in predictive power, but rather their role as a high-throughput, objective, and reproducible alternative for the assessment of digitized biopsies. This is particularly relevant because the VALIGA cohort includes centralized histopathology scoring to reduce measurement error, which is not possible in real world practice where interobserver variability based on local pathology scores can be substantial.^14^

This study has several important limitations. Our findings are primarily generalizable to European populations, as over 97% of the VALIGA cohort were White. Thus, analyses regarding the ethnicity-adjusted model are limited in their generalizability. Given the known ethnic differences in the morphology and disease progression of IgAN,^5^^,^^34^ validation of pathomics in diverse cohorts, including Asian and North American populations, will be essential. Second, although the centralized scoring in the VALIGA cohort provides a robust reference standard, we did not undertake external validation in independent cohorts, which remains essential to further establish the model's generalizability. Last, the retrospective analysis of the cohort limits the evaluation of the clinical impact of pathomics in real-world clinical settings. Prospective studies, study populations receiving recently approved treatments, and integrative frameworks using other novel biomarkers including transcriptomics are warranted to assess the clinical utility in the future.

Overall, our study provides a proof-of-concept and a blueprint for the integration of pathomics into established risk prediction models in nephrology.

## Appendix

### The List of Members of the International IgA Nephropathy Network are as Follows:

VALIGA investigators: M.L. Russo (Fondazione Ricerca Molinette, Torino, Italy); S. Troyanov (Division of Nephrology, Department of Medicine, Hopital du Sacre-Coeur de Montreal, Montreal, Quebec, Canada); H.T. Cook (Centre for Complement and Inflammation Research, Department of Medicine, Imperial College, London, England); I.S.D. Roberts (Department of Cellular Pathology, Oxford University Hospitals NHS Foundation Trust, John Radcliffe Hospital, Oxford, United Kingdom); V. Tesar, (Department of Nephrology, 1st Faculty of Medicine and General University Hospital, Charles University, Prague, Czech Republic); D. Maixnerova (Department of Nephrology, 1st Faculty of Medicine and General University Hospital, Charles University, Prague, Czech Republic); S. Lundberg (Nephrology Unit, Department of Clinical Sciences, Karolinska Institute, Stockholm, Sweden); L. Gesualdo (Department of Nephrology, Emergency and Organ Transplantation, University of Bari “Aldo Moro,” Foggia-Bari, Italy); F. Emma (Division of Nephrology, Department of Pediatric Subspecialties, Bambino Gesù Children’s Hospital IRCCS, Rome, Italy); F. Diomedi (Division of Nephrology, Department of Pediatric Subspecialties, Bambino Gesù Children’s Hospital IRCCS, Rome, Italy); G. Beltrame (Nephrology and Dialysis Unit, San Giovanni Bosco Hospital, and University of Turin, Turin, Italy); C. Rollino (Nephrology and Dialysis Unit, San Giovanni Bosco Hospital, and University of Turin, Turin, Italy); A. Amore (Nephrology Unit, Regina Margherita Children’s Hospital, Turin, Italy); R. Camilla (Nephrology Unit, Regina Margherita Children’s Hospital, Turin, Italy); L. Peruzzi (Nephrology Unit, Regina Margherita Children’s Hospital, Turin, Italy); M. Praga (Nephrology Unit, Hospital 12 de Octubre, Madrid, Spain); S. Feriozzi (Nephrology Unit, Belcolle Hospital, Viterbo, Italy), R. Polci, (Nephrology Unit, Belcolle Hospital, Viterbo, Italy); G. Segoloni, (Division of Nephrology Dialysis and Transplantation, Department of Medical Sciences, Città della Salute e della Scienza Hospital and University of Turin, Turin, Italy); L. Colla (Division of Nephrology Dialysis and Transplantation, Department of Medical Sciences, Città della Salute e della Scienza Hospital and University of Turin, Turin, Italy); A. Pani (Nephrology Unit, G. Brotzu Hospital, Cagliari, Italy); D. Piras (Nephrology Unit, G. Brotzu Hospital, Cagliari, Italy), A. Angioi (Nephrology Unit, G. Brotzu Hospital, Cagliari, Italy); G. Cancarini, (Nephrology Unit, Spedali Civili University Hospital, Brescia, Italy); S. Ravera (Nephrology Unit, Spedali Civili University Hospital, Brescia, Italy); M. Durlik (Department of Transplantation Medicine, Nephrology, and Internal Medicine, Medical University of Warsaw, Warsaw, Poland); E. Moggia (Nephrology Unit, Santa Croce Hospital, Cuneo, Italy); J. Ballarin (Department of Nephrology, Fundacion Puigvert, Barcelona, Spain); S. Di Giulio (Nephrology Unit, San Camillo Forlanini Hospital, Rome, Italy); F. Pugliese (Department of Nephrology, Policlinico Umberto I University Hospital, Rome, Italy); I. Serriello (Department of Nephrology, Policlinico Umberto I University Hospital, Rome, Italy); Y. Caliskan (Division of Nephrology, Department of Internal Medicine, Istanbul Faculty of Medicine, Istanbul University, Istanbul, Turkey); M. Sever (Division of Nephrology, Department of Internal Medicine, Istanbul Faculty of Medicine, Istanbul University, Istanbul, Turkey); I. Kilicaslan (Department of Pathology, Istanbul Faculty of Medicine, Istanbul University, Istanbul, Turkey); F. Locatelli (Department of Nephrology and Dialysis, Alessandro Manzoni Hospital, ASST Lecco, Italy); L. Del Vecchio (Department of Nephrology and Dialysis, Alessandro Manzoni Hospital, ASST Lecco, Italy); J.F.M. Wetzels (Departments of Nephrology, Radboud University Medical Center, Nijmegen, the Netherlands); H. Peters (Departments of Nephrology, Radboud University Medical Center, Nijmegen, the Netherlands); U. Berg (Division of Pediatrics, Department of Clinical Science, Intervention and Technology, Huddinge, Sweden); F. Carvalho (Nephrology Unit, Hospital de Curry Cabral, Lisbon, Portugal); A.C. da Costa Ferreira (Nephrology Unit, Hospital de Curry Cabral, Lisbon, Portugal); M. Maggio (Nephrology Unit, Hospital Maggiore di Lodi, Lodi, Italy); A. Wiecek (Department Nephrology, Endocrinology and Metabolic Diseases, Silesian University of Medicine, Katowice, Poland); M. Ots-Rosenberg (Nephrology Unit, Tartu University Clinics, Tartu, Estonia); R. Magistroni (Department of Nephrology, Policlinic of Modena and Reggio Emilia; Modena, Italy); R. Topaloglu (Department of Pediatric Nephrology and Rheumatology, Hacettepe University, Ankara, Turkey); Y. Bilginer (Department of Pediatric Nephrology and Rheumatology, Hacettepe University, Ankara, Turkey); M. D’Amico (Nephrology Unit, S. Anna Hospital, Como, Italy); M. Stangou (Department of Nephrology, Hippokration General Hospital, Aristotle University of Thessaloniki, Thessaloniki, Greece); F. Giacchino (Nephrology Unit, Ivrea Hospital, Ivrea, Italy); D. Goumenos (Department of Nephrology, University Hospital of Patras, Patras, Greece); P. Kalliakmani (Department of Nephrology, University Hospital of Patras, Patras, Greece); M. Papasotiriou (Department of Nephrology, University Hospital of Patras, Patras, Greece); K. Galesic (Department of Nephrology, University Hospital Dubrava, Zagreb, Croatia); C. Geddes (Renal Unit, Western Infirmary Glasgow, Glasgow, United Kingdom); K. Siamopoulos (Nephrology Unit, Medical School University of Ioanina, Ioannina, Greece); O. Balafa (Nephrology Unit, Medical School University of Ioanina, Ioannina, Greece); M. Galliani (Nephrology Unit, S.Pertini Hospital, Rome, Italy); P. Stratta (Department of Nephrology, Maggiore della Carità Hospital, Piemonte Orientale University, Novara, Italy); M. Quaglia (Department of Nephrology, Maggiore della Carità Hospital, Piemonte Orientale University, Novara, Italy); R. Bergia (Nephrology Unit, Degli Infermi Hospital, Biella, Italy); R. Cravero (Nephrology Unit, Degli Infermi Hospital, Biella, Italy); M. Salvadori, (Department of Nephrology, Careggi Hospital, Florence, Italy); L. Cirami (Department of Nephrology, Careggi Hospital, Florence, Italy); B. Fellstrom (Renal Department, University of Uppsala, Uppsala, Sweden); H. Kloster Smerud (Renal Department, University of Uppsala, Uppsala, Sweden); F. Ferrario (Nephropathology Unit, San Gerardo Hospital, Monza, Italy); T. Stellato (Nephropathology Unit, San Gerardo Hospital, Monza, Italy); J. Egido (Department of Nephrology, Fundacion Jimenez Diaz, Madrid, Spain); C. Martin (Department of Nephrology, Fundacion Jimenez Diaz, Madrid, Spain); J. Floege (Nephrology and Immunology, Medizinische Klinik II, University of Aachen, Aachen, Germany); F. Eitner (Nephrology and Immunology, Medizinische Klinik II, University of Aachen, Aachen, Germany); A. Lupo (Department of Nephrology, University of Verona, Verona, Italy); P. Bernich (Department of Nephrology, University of Verona, Verona, Italy); P. Menè (Department of Nephrology,S. Andrea Hospital, Rome, Italy); M. Morosetti (Nephrology Unit, Grassi Hospital, Ostia, Italy); C. van Kooten, (Department of Nephrology, Leiden University Medical Centre, Leiden, The Netherlands); T. Rabelink (Department of Nephrology, Leiden University Medical Centre, Leiden, The Netherlands); M.E.J. Reinders (Department of Nephrology, Leiden University Medical Centre, Leiden, The Netherlands); J.M. Boria Grinyo (Department of Nephrology, Hospital Bellvitge, Barcelona, Spain); S. Cusinato (Nephrology Unit, Borgomanero Hospital, Borgomanero, Italy); L. Benozzi (Nephrology Unit, Borgomanero Hospital, Borgomanero, Italy); S. Savoldi, (Nephrology Unit, Civile Hospital, Ciriè, Italy); C. Licata (Nephrology Unit, Civile Hospital, Ciriè, Italy); M. Mizerska-Wasiak (Department of Pediatrics, Medical University of Warsaw, Warsaw, Poland); G. Martina (Nephrology Unit, Chivasso Hospital, Chivasso, Italy); A. Messuerotti (Nephrology Unit, Chivasso Hospital, Chivasso, Italy); A. Dal Canton (Nephrology Unit, S. Matteo Hospital, Pavia, Italy); C. Esposito (Nephrology Unit, Maugeri Foundation, Pavia, Italy); C. Migotto (Nephrology Unit, Maugeri Foundation, Pavia, Italy); G. Triolo (Nephrology Unit CTO, Turin, Italy); F. Mariano (Nephrology Unit CTO, Turin, Italy); C. Pozzi (Nephrology Unit, Bassini Hospital, Cinisello Balsamo, Italy); R. Boero (Nephrology Unit, Martini Hospital, Turin, Italy).

VALIGA pathology investigators: S. Bellur (Department of Cellular Pathology, Oxford University Hospitals NHS Foundation Trust, John Radcliffe Hospital, Oxford, United Kingdom); G. Mazzucco (Pathology Department, University of Turin, Turin, Italy); C. Giannakakis (Pathology Department, La Sapienza University, Rome, Italy); E. Honsova (Department of Clinical and Transplant Pathology, Institute for Clinical and Experimental Medicine, Prague, Czech Republic); B. Sundelin (Department of Pathology and Cytology, Karolinska University Hospital, Karolinska Institute, Stockholm, Sweden); A.M. Di Palma (Nephrology Unit, Aldo Moro University, Foggia-Bari, Italy); F. Ferrario (Nephropathology Unit, San Gerardo Hospital, Monza, Italy); E. Gutiérrez (Renal, Vascular and Diabetes Research Laboratory, Fundación Instituto de Investigaciones Sanitarias-Fundación Jiménez Díaz, Universidad Autónoma de Madrid, Madrid, Spain); A.M. Asunis (Department of Pathology, Brotzu Hospital, Cagliari, Italy); J. Barratt (The John Walls Renal Unit, Leicester General Hospital, Leicester, United Kingdom); R. Tardanico (Department of Pathology, Spedali Civili Hospital, University of Brescia, Brescia, Italy); A. Perkowska-Ptasinska (Department of Transplantation Medicine, Nephrology and Internal Medicine, Medical University of Warsaw, Warsaw, Poland); J. Arce Terroba (Pathology Department, Fundació Puigvert, Barcelona, Spain); M. Fortunato (Pathology Department, S. Croce Hospital, Cuneo, Italy); A. Pantzaki (Department of Pathology, Hippokration Hospital, Thessaloniki, Greece); Y. Ozluk (Department of Pathology, Istanbul University, Istanbul Faculty of Medicine, Istanbul, Turkey); E. Steenbergen (Radboud University Medical Center, Department of Pathology, Nijmegen, The Netherlands); M. Soderberg (Department of Pathology, Drug Safety and Metabolism, Huddinge, Sweden); Z. Riispere (Department of Pathology, University of Tartu, Tartu, Estonia); L. Furci (Pathology Department, University of Modena, Italy); D. Orhan (Department of Pediatrics, Division of Rheumatology, Hacettepe University Faculty of Medicine, Ankara, Turkey); D. Kipgen (Pathology Department, Queen Elizabeth University Hospital, Glasgow, United Kingdom); D. Casartelli (Pathology Department, Manzoni Hospital, Lecco, Italy); D. Galesic Ljubanovic (Nephrology Department, University Hospital, Zagreb, Croatia; Zagreb, Croatia); H Gakiopoulou (Department of Pathology, National and Kapodistrian University of Athens, Athens, Greece); E. Bertoni (Nephrology Department, Careggi Hospital, Florence, Italy); P. Cannata Ortiz (Pathology Department, IIS-Fundacion Jimenez Diaz UAM, Madrid, Spain); H. Karkoszka (Nephrology, Endocrinology and Metabolic Diseases, Medical University of Silesia, Katowice, Katowice, Poland); H.J. Groene (Cellular and Molecular Pathology, German Cancer Research Center, Heidelberg, Germany); A. Stoppacciaro (Surgical Pathology Units, Department of Clinical and Molecular Medicine, Ospedale Sant’Andrea, Sapienza University of Rome, Rome, Italy); I. Bajema (Department of Pathology, Leiden University Medical Center, Leiden, The Netherlands); J. Bruijn (Department of Pathology, Leiden University Medical Center, Leiden, The Netherlands); X. Fulladosa Oliveras (Nephrology Unit, Bellvitge University Hospital, Hospitalet de Llobregat, Barcelona, Spain); J. Maldyk (Division of Pathomorphology, Children’s Clinical Hospital, Medical University of Warsaw, Warsaw, Poland); and E. Ioachim (Department of Pathology, Medical School, University of Ioannina, Ioannina, Greece); the Oxford derivation and North American validation investigators: Bavbek N (Department of Pathology, Vanderbilt University, Nashville, Tennessee); Cook T (Imperial College, London, England), Troyanov S (Division of Nephrology, Department of Medicine, Hopital du Sacre-Coeur de Montreal, Montreal, Quebec, Canada); Alpers C (Department of Pathology, University of Washington Medical Center, Seattle, Washington), Amore A (Nephrology, Dialysis and Transplantation Unit, Regina Margherita Children’s Hospital, University of Turin, Turin, Italy), Barratt J (The John Walls Renal Unit, Leicester General Hospital, Leicester, England); Berthoux F (Department of Nephrology, Dialysis, and Renal Transplantation, Hôpital Nord, CHU de Saint-Etienne, Saint-Etienne, France); Bonsib S (Department of Pathology, LSU Health Sciences Center, Shreveport, Los Angeles); Bruijn J (Department of Pathology, Leiden University Medical Center, Leiden, The Netherlands); D’Agati V (Department of Pathology, Columbia University College of Physicians & Surgeons, New York, New York); D’Amico G (Fondazione D’Amico per la Ricerca sulle Malattie Renali, Milan, Italy); Emancipator S (Department of Pathology, Case Western Reserve University, Cleveland, Ohio); Emmal F (Division of Nephrology and Dialysis, Department of Nephrology and Urology, Bambino Gesù Children’s Hospital and Research Institute, Piazza S Onofrio, Rome, Italy); Ferrario F (Renal Immunopathology Center, San Carlo Borromeo Hospital, Milan, Italy); Fervenza F (Division of Nephrology and Hypertension, Mayo Clinic, Rochester); Florquin S (Department of Pathology, Academic Medical Center, University of Amsterdam, Amsterdam, The Netherlands); Fogo A (Department of Pathology, Vanderbilt University, Nashville, Tennessee); Geddes C (The Renal Unit, Western Infirmary, Glasgow, Scotland); Groene H (Department of Cellular and Molecular Pathology, German Cancer Research Center, Heidelberg, Germany); Haas M (Department of Pathology and Laboratory Medicine, Cedars-Sinai Medical Center, Los Angeles, California); Hill P (St Vincent’s Hospital, Melbourne, Australia); Hogg R (Scott and White Medical Center, Temple, Texas (retired)); Hsu S (Division of Nephrology, Hypertension and Renal Transplantation, College of Medicine, University of Florida, Gainesville, Florida); Hunley T (Department of Pathology, Vanderbilt University, Nashville, Tennessee); Hladunewich (Division of Nephrology, Sunnybrook Health Science Center, University of Toronto, Ontario, Canada M); Jennette C (Department of Pathology and Laboratory Medicine, University of North Carolina, Chapel Hill, North Carolina); Joh K (Division of Immunopathology, Clinical Research Center Chiba, East National Hospital, Chiba, Japan); Julian B (Department of Medicine, University of Alabama at Birmingham, Birmingham, Alabama); Kawamura T (Division of Nephrology and Hypertension, Jikei University School of Medicine, Tokyo, Japan); Lai F (The Chinese University of Hong Kong, Hong Kong); Leung C (Department of Medicine, Prince of Wales Hospital, Chinese University of Hong Kong, Hong Kong); Li L (Research Institute of Nephrology, Jinling Hospital, Nanjing University School of Medicine, Nanjing, China); Li P (Department of Medicine, Prince of Wales Hospital, Chinese University of Hong Kong, Hong Kong); Liu Z (Research Institute of Nephrology, Jinling Hospital, Nanjing University School of Medicine, Nanjing, China); Massat A (Division of Nephrology and Hypertension, Mayo Clinic, Rochester, Minnesota); Mackinnon B (The Renal Unit, Western Infirmary, Glasgow, Scotland); Mezzano S (Departamento de Nefrología, Escuela de Medicina, Universidad 5 Austral, Valdivia, Chile); Schena F (Renal, Dialysis and Transplant Unit, Policlinico, Bari, Italy); Tomino Y (Division of Nephrology, Department of Internal Medicine, Juntendo University School of Medicine, Tokyo, Japan); Walker P (Nephropathology Associates, Little Rock, Arkansas); Wang H (Renal Division of Peking University First Hospital, Peking University Institute of Nephrology, Beijing, China (deceased)); Weening J (Erasmus Medical Center, Rotterdam, The Netherlands); and Yoshikawa N (Department of Pediatrics, Wakayama Medical University, Wakayama City, Japan).

International investigators: Cai-Hong Zeng (Nanjing University School of Medicine, Nanjing, China); Sufang Shi (Peking University Institute of Nephrology, Beijing, China); C. Nogi (Juntendo University, Faculty of Medicine, Tokyo, Japan); H. Suzuki (Juntendo University, Faculty of Medicine, Tokyo, Japan); K. Koike (Division of Nephrology and Hypertension, Department of Internal Medicine, Jikei University School of Medicine, Tokyo, Japan); K. Hirano (Division of Nephrology and Hypertension, Department of Internal Medicine, Jikei University School of Medicine, Tokyo, Japan); T. Kawamura (Division of Nephrology and Hypertension, Department of Internal Medicine, Jikei University School of Medicine, Tokyo, Japan); T. Yokoo (Division of Nephrology and Hypertension, Department of Internal Medicine, Jikei University School of Medicine, Tokyo, Japan); M. Hanai (Division of Nephrology, Department of Medicine, Kurume University School of Medicine, Fukuoka, Japan); K. Fukami (Division of Nephrology, Department of Medicine, Kurume University School of Medicine, Fukuoka, Japan); K. Takahashi (Department of Nephrology, Fujita Health University School of Medicine, Aichi, Japan); Y. Yuzawa (Department of Nephrology, Fujita Health University School of Medicine, Aichi, Japan); M. Niwa (Department of Nephrology, Nagoya University Graduate School of Medicine, Aichi, Japan); Y. Yasuda (Department of Nephrology, Nagoya University Graduate School of Medicine, Aichi, Japan); S. Maruyama (Department of Nephrology, Nagoya University Graduate School of Medicine, Aichi, Japan); D. Ichikawa (Division of Nephrology and Hypertension, Department of Internal Medicine, St Marianna University School of Medicine, Kanagawa, Japan); T. Suzuki (Division of Nephrology and Hypertension, Department of Internal Medicine, St Marianna University School of Medicine, Kanagawa, Japan); S. Shirai (Division of Nephrology and Hypertension, Department of Internal Medicine, St Marianna University School of Medicine, Kanagawa, Japan); A. Fukuda (First Department of Internal Medicine, Faculty of Medicine, University of Miyazaki, Miyazaki, Japan); S. Fujimoto (Department of Hemovascular Medicine and Artificial Organs, Faculty of Medicine, University of Miyazaki, Miyazaki, Japan); H. Trimarchi (Division of Nephrology, Hospital Britanico, Buenos Aires, Argentina).

## Disclosure

PB received consulting and lecture fees from CSL Vifor, Otsuka, Novartis, Bristol Myers Squibb, Roche, Novo Nordisk, Astellas, and aetherAI, none of which was relevant to this work. SB reports consulting fees from Roche, Novartis Biogen, Vera, Eledon, HiBio, and research funding from Novartis and BioCryst; none of which was relevent to this work. The other authors declared no competing interests.

## Supplementary Material

Supplementary file (PDF) **Figure S1.** Cohort refinement process. **Figure S2.** Explained variance by clusters of pathomics variables. **Figure S3.** Associations between pathomics variables, estimated glomerular function and proteinuria at time of kidney biopsy. **Table S1.** Description of pathomics variables. **Table S2.** STROBE checklist for cohort studies. **Table S3.** Comparison of patient characteristics across subcohorts. **Table S4.** Clustering of pathomics variables. **Table S5.** Measures of predictive performance for models with MEST-C and pathomics.

## Footnotes

Supplementary file (PDF)
**Figure S1.** Cohort refinement process. **Figure S2.** Explained variance by clusters of pathomics variables. **Figure S3.** Associations between pathomics variables, estimated glomerular function and proteinuria at time of kidney biopsy. **Table S1.** Description of pathomics variables. **Table S2.** STROBE checklist for cohort studies. **Table S3.** Comparison of patient characteristics across subcohorts. **Table S4.** Clustering of pathomics variables. **Table S5.** Measures of predictive performance for models with MEST-C and pathomics.
