# Supplementary material for: Incorporating Pathomics Into IgAN Risk Prediction
Source: Kidney Int Rep. 2026 Jul 22;11(10):106708. doi: 10.1016/j.ekir.2026.106708 (PMC13519253; doi:10.1016/j.ekir.2026.106708)

Supplementary Material to: Incorporating pathomics into IgA nephropathy risk prediction

David L. Hölscher[1,2,$], Sean J. Barbour[3,4,$,*], Rosanna Coppo[5,6], Lee Er[3], Roman D. Bülow[1], Martin Strauch[1], Vladimir Tesar[7], Jonathan Barratt[8,9], Ian S. D. Roberts[10], Peter Boor[1,2,*] for the International IgA Nephropathy Networkϕ

1 Institute of Pathology, RWTH Aachen University Hospital, Aachen, Germany
2 Department of Nephrology and Immunology, RWTH Aachen University Hospital, Aachen, Germany
3 University of British Columbia, Division of Nephrology, Vancouver, Canada
4 BC Renal, Vancouver, Canada
5 Fondazione Ricerca Molinette, Torino, Italy
6 Regina Margherita Children's University Hospital, Torino, Italy
7 Department of Nephrology, 1st Faculty of Medicine and General University Hospital, Charles University, Prague, Czech Republic
8 John Walls Renal Unit, University Hospital of Leicester National Health Service Trust, Leicester, United Kingdom
9 Department of Cardiovascular Sciences, University of Leicester, Leicester, United Kingdom
10 Department of Cellular Pathology, Oxford University Hospitals National Health Services Foundation Trust, Oxford, United Kingdom

ϕ International IgAN Network contributor information are provided in the appendix
$ These authors contributed equally
* Address correspondence to:

Sean Barbour, M.D. MSc
Division of Nephrology
University of British Columbia
5th floor, 2775 Laurel Street
Vancouver, BC, V5Z 1M9
Phone: + 1 604-875-5950
E-Mail: sean.barbour@vch.ca

Peter Boor, M.D., Ph.D.
Institute of Pathology
RWTH Aachen University Hospital
Pauwelsstrasse 30
52074 Aachen, Germany
Phone: +49 241 80 85227
E-Mail: pboor@ukaachen.de

Table of Contents

- Supplementary Table S1: Description of pathomics variables
- Supplementary Table S2: STROBE checklist for cohort studies
- Supplementary Table S3: Comparison of patient characteristics across subcohorts
- Supplementary Table S4: Clustering of pathomics variables
- Supplementary Table S5: Measures of predictive performance for models with MEST-C and pathomics
- Supplementary Figure S1: Cohort refinement process
- Supplementary Figure S2: Explained variance by clusters of pathomics variables
- Supplementary Figure S3: Associations between pathomics variables, estimated glomerular function and proteinuria at time of kidney biopsy

Supplementary Table S1. Description of the pathomics variables. All pathomics variables except for area percentages are summarized per patient using the median value (interquartile range) of the distribution.

Pathomics variables	Description	Value in the analytic cohort
**Arterial variables**		
Diameter [µm]	Diameter of the largest circle fitting into the segmented artery including its lumen	15.57 (14.31, 17.36)
Distance [µm]	Distance between the border of the instance and the closest neighboring instance	3.21 (2.50, 4.52)
Lumen diameter [µm]	Diameter of the largest circle fitting into the segmented lumen of the artery	4.72 (3.37, 5.72)
Lumen area [µm²]	Area of the cross-section of the segmented artery lumen	39.12 (23.42,55.55)
Wall diameter [µm]	Measuring the arterial wall thickness of the segmented artery without its lumen (including the intimal and media layer)	5.73 (5.12, 6.36)
Wall area [µm²]	Area of the cross-section of the segmented artery without its lumen (including the intimal and media layer)	287.60 (236.52, 373.54)
**Glomerular variables (including Bowman's capsule)**		
Area [µm²]	Area of the cross-section of the segmented glomerulus including the Bowman's capsule	13704.00 (9909.77, 17625.14)
Bowman area [µm²]	Area of the cross-section of the segmented glomerulus including the Bowman's capsule but without the glomerular tuft	5770.24 (4812.23, 6938.51)
Diameter [µm]	Diameter of the largest circle fitting into the segmented Glomerulus including the Bowman's capsule	105.88 (87.46, 122.61)
Distance [µm]	Distance between the border of the instance and the closest neighboring instance of the same class	171.17 (121.94, 222.93)
Circularity	Ratio of the segmented area multiplied by 4π to its squared perimeter, measuring how circular the glomerulus is	0.56 (0.47, 0.63)
Eccentricity	Ratio of the distance between the segmented glomerular focal points over the major axis' length	0.64 (0.60, 0.68)

Elongation	Function of the length of the segmented glomerular minor and major axis	0.23 (0.20, 0.27)
Solidity	Ratio of the glomerular area to the area of its convex hull, measuring the density of the glomerulus	0.94 (0.92, 0.96)
**Glomerular tuft variables**		
Area [μm²]	Area of the cross-section of the segmented glomerular tuft	7234.04 (3102.65, 10742.92)
Tuft-to-capsule-ratio	Ratio between the size of the glomerular tuft and the size of the full glomerulus including its capsule	0.57 (0.43, 0.64)
Circularity	Ratio of the segmented area multiplied by 4π to its squared perimeter, measuring how circular the tuft is	0.37 (0.33, 0.42)
Eccentricity	Ratio of the distance between the segmented glomerular tuft's focal points over the major axis' length	0.67 (0.62, 0.71)
Elongation	Function of the length of the segmented glomerular tuft's minor and major axis	0.26 (0.22, 0.30)
Solidity	Ratio of the glomerular tuft area to the area of its convex hull, measuring the density of the glomerular tuft	0.89 (0.87, 0.91)
**Tubular variables**		
Area [μm²]	Area of the cross-section of the segmented tubule	1404.16 (1103.33, 1781.39)
Diameter [μm]	Diameter of the largest circle fitting inside the segmented tubule	30.05 (27.03, 33.38)
Distance [μm]	Distance between the border of the instance and the closest neighboring instance	2.38 (1.97, 3.02)
Weighted mean tubular area [%]	Weighted mean of the segmented area percentage of all tubules adjusted for the overall area of the segmented tissue	47.97 (40.93, 55.05)
**Other variables**		
Weighted mean interstitial area [%]	Weighted mean of the segmented area percentage of the interstitial background adjusted for the overall area of the segmented tissue	44.59 (36.94, 52.25)

Supplementary Table S2. The Strengthening the Reporting of Observational Studies in Epidemiology (STROBE) checklist for cohort studies.

	**Item No.**	**Recommendation**	**Page No.**
Title and abstract	1	(*a*) Indicate the study's design with a commonly used term in the title or the abstract	1-2
		(*b*) Provide in the abstract an informative and balanced summary of what was done and what was found	2-3
Introduction			
Background/ rationale	2	Explain the scientific background and rationale for the investigation being reported	4-5
Objectives	3	State specific objectives, including any prespecified hypotheses	4-5
Methods			
Study design	4	Present key elements of study design early in the paper	5-6
Setting	5	Describe the setting, locations, and relevant dates, including periods of recruitment, exposure, follow-up, and data collection	5
Participants	6	(*a*) Give the eligibility criteria, and the sources and methods of selection of participants. Describe methods of follow-up	5
		(*b*) For matched studies, give matching criteria and number of exposed and unexposed	n/a
Variables	7	Clearly define all outcomes, exposures, predictors, potential confounders, and effect modifiers. Give diagnostic criteria, if applicable	5-6
Data sources/ measurement	8*	For each variable of interest, give sources of data and details of methods of assessment (measurement). Describe comparability of assessment methods if there is more than one group	5-6
Bias	9	Describe any efforts to address potential sources of bias	6
Study size	10	Explain how the study size was arrived at	5, Supp. Figure 1
Quantitative variables	11	Explain how quantitative variables were handled in the analyses. If applicable, describe which groupings were chosen and why	5-6
Statistical methods	12	(*a*) Describe all statistical methods, including those used to control for confounding	7-9
		(*b*) Describe any methods used to examine subgroups and interactions	7-9
		(*c*) Explain how missing data were addressed	9
		(*d*) If applicable, explain how loss to follow-up was addressed	7
		(*e*) Describe any sensitivity analyses	n/a
Results			
Participants	13*	(a) Report numbers of individuals at each stage of study-eg numbers potentially eligible, examined for eligibility, confirmed eligible, included in the study, completing follow-up, and analyzed	10, Supp. Figure 1
		(b) Give reasons for non-participation at each stage	5, 10, Supp. Figure 1

		(c) Consider use of a flow diagram	Supp. Figure 1
Descriptive data	14*	(a) Give characteristics of study participants (eg demographic, clinical, social) and information on exposures and potential confounders	10, Table 1
		(b) Indicate number of participants with missing data for each variable of interest	9
		(c) Summarize follow-up time (eg, average and total amount)	10
Outcome data	15*	Report numbers of outcome events or summary measures over time	10

Supplementary Table S3. Comparison of patient characteristics in the overall VALIGA cohort, the included patients (meeting all inclusion criteria along with available whole-slide images (WSIs)) and the excluded patients which were excluded due to unavailable WSIs. A Cohen's d < 0.1 indicates a negligible difference between groups.

	**VALIGA (overall)**	**Included patients (with available WSIs)**	**Excluded patients (due to un-available WSIs)**	**Absolute standardized difference (Cohen's d)**
Number of patients	954	555	399	N/A
Duration of follow-up (years)	7.5 [4.3, 11.1]	7.3 [4.2, 11.1]	7.5 [4.8, 11.1]	0.070
Year of biopsy	2004 [2000, 2007]	2004 [2000, 2007]	2004 [2000, 2007]	0.021
Age (years)	38.7 [28.8, 50.0]	37.9 [28.3, 48.6]	39.4 [29.5, 54.7]	0.152
Male sex	692 (72.5%)	402 (72.4%)	290 (72.7%)	0.006
Ethnicity:				0.064
White	932 (97.7%)	540 (97.3%)	392 (98.2%)	
Other	22 (2.3%)	15 (2.7%)	7 (1.8%)	
Creatinine at biopsy (μmol/L)	106.1 [85.0, 148.0]	106.1 [86.0, 148.0]	106.1 [81.3, 150.0]	0.031
eGFR at biopsy (ml/min/1.73m2)	71.0 [46.2, 94.9]	71.8 [46.3, 94.7]	68.7 [45.8, 96.4]	0.006
MAP at biopsy (mmHg)	99.3 [93.3, 106.7]	98.3 [92.3, 106.7]	100.0 [93.3, 106.7]	0.080
Proteinuria at biopsy (g/day)	1.3 [0.7, 2.6]	1.3 [0.7, 2.6]	1.3 [0.6, 2.6]	0.005
Pathology:				
M1	275 (28.8%)	165 (29.7%)	110 (27.6%)	0.048
E1	96 (10.1%)	59 (10.6%)	37 (9.3%)	0.045
S1	713 (74.7%)	433 (78%)	280 (70.2%)	0.180
T1	187 (19.6%)	121 (21.8%)	66 (16.5%)	0.127
T2	38 (4%)	21 (3.8%)	17 (4.3%)	
Crescents	95 (10%)	54 (9.7%)	41 (10.3%)	0.018
Use of RASi at biopsy	429 (45%)	246 (44.3%)	183 (45.9%)	0.031
Use of RASi after biopsy	867 (91.2%)	509 (91.7%)	358 (90.4%)	0.046
IS prior to biopsy	125 (13.1%)	69 (12.4%)	56 (14%)	0.047
IS after biopsy	392 (41.1%)	217 (39.1%)	175 (43.9%)	0.097

Supplementary Table S4. The results of the clustering analysis applied to the pathomics variables. The pathomics variables with the smallest 1-R^2 ratio have the strongest correlation with other variables in the same cluster and the least correlation with variables in other clusters and are indicated in red.
*Due to rounding the 1-R^2 ratios appear similar.

Cluster	Pathomics variable	R^2 with own cluster	R^2 with next closest cluster	1-R^2 ratio
**1**	Tubular diameter	0.89	0.26	**0.15**
	Tubular area	0.84	0.28	0.22
	Weighted mean tubular area	0.86	0.41	0.23
	Weighted mean interstitial area	0.87	0.48	0.25
**2**	Tuft eccentricity	0.99	0.39	**0.01***
	Tuft elongation	0.99	0.40	0.01
**3**	Arterial diameter	0.90	0.26	**0.13**
	Arterial wall area	0.84	0.20	0.19
	Arterial wall diameter	0.83	0.09	0.19
**4**	Glomerular area	0.89	0.30	0.15
	Glomerular diameter	0.88	0.37	0.19
	Tuft area	0.95	0.29	**0.07**
	Tuft-to-capsule-ratio	0.58	0.32	0.61
**5**	Glomerular circularity	0.82	0.35	**0.28**
	Glomerular solidity	0.76	0.34	0.36
	Tuft circularity	0.75	0.21	0.32
	Tuft solidity	0.75	0.25	0.34
**6**	Arterial lumen area	0.86	0.17	0.17
	Arterial lumen diameter	0.86	0.07	**0.15**
**7**	Glomerular distance	1.00	0.05	**0.00**
**8**	Tubular distance	0.79	0.39	0.34
	Arterial distance	0.79	0.26	**0.28**
**9**	Bowman area	1.00	0.11	**0.00**
**10**	Glomerular eccentricity	0.99	0.39	**0.01***
	Glomerular elongation	0.99	0.40	0.01

Supplementary Table S5. Measures of prediction performance after internal validation for the multivariable models of MEST-C and pathomics variables with or without clinical predictors. Apparent results are those from the primary analysis. Optimism-corrected results are based on bootstrap resampling to estimate the optimism that results from evaluating model performance in the same cohort in which the model was derived. AIC and R^2_D are measures of model fit, with lower and higher values respectively indicating better model fit. The C-statistic was evaluated at a 5-year time horizon.

**Measure of prediction performance**	**Model with MEST-C**		**Model with pathomics**	
	Apparent	Optimism-corrected	Apparent	Optimism-corrected
**Models without clinical variables**				
R^2_D	12.9%	11.1%	13.0%	9.1%
C-statistic (95% CI)	0.69 (0.68, 0.70)	0.70 (0.69, 0.71)	0.74 (0.73, 0.75)	0.74 (0.73, 0.75)
**Models with clinical variables**				
R^2_D	27.0%	24.5%	27.6%	23.4%
C-statistic (95% CI)	0.79 (0.78, 0.81)	0.79 (0.78, 0.81)	0.76 (0.75, 0.77)	0.76 (0.75, 0.77)

Supplementary Figure S1. Derivation of the analytic cohort. IIgAN-PT, International IgA Nephropathy Prediction Tool.

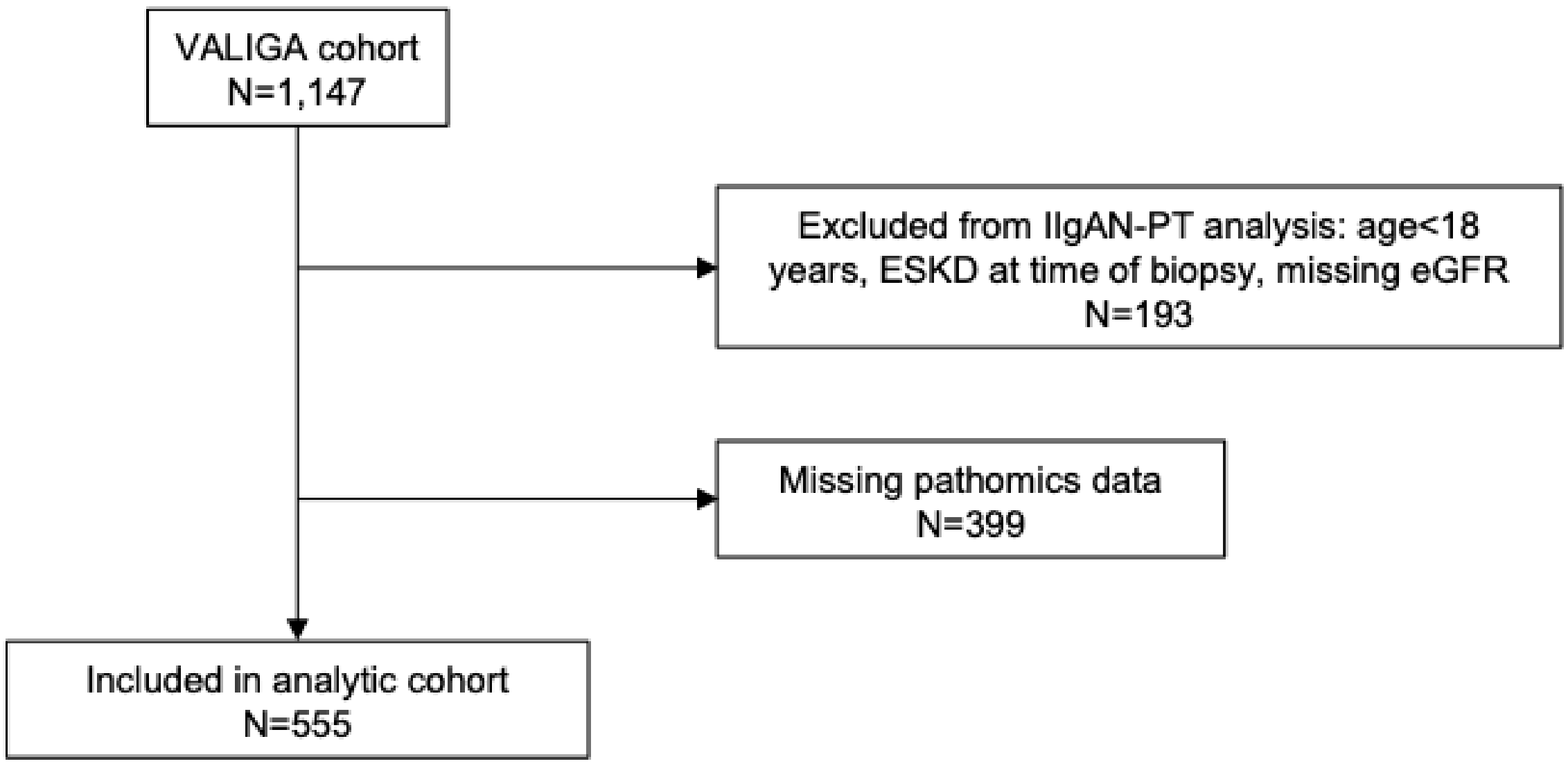

Supplementary Figure S2. A graphical representation of the explained variance from the clustering analysis applied to the pathomics variables.

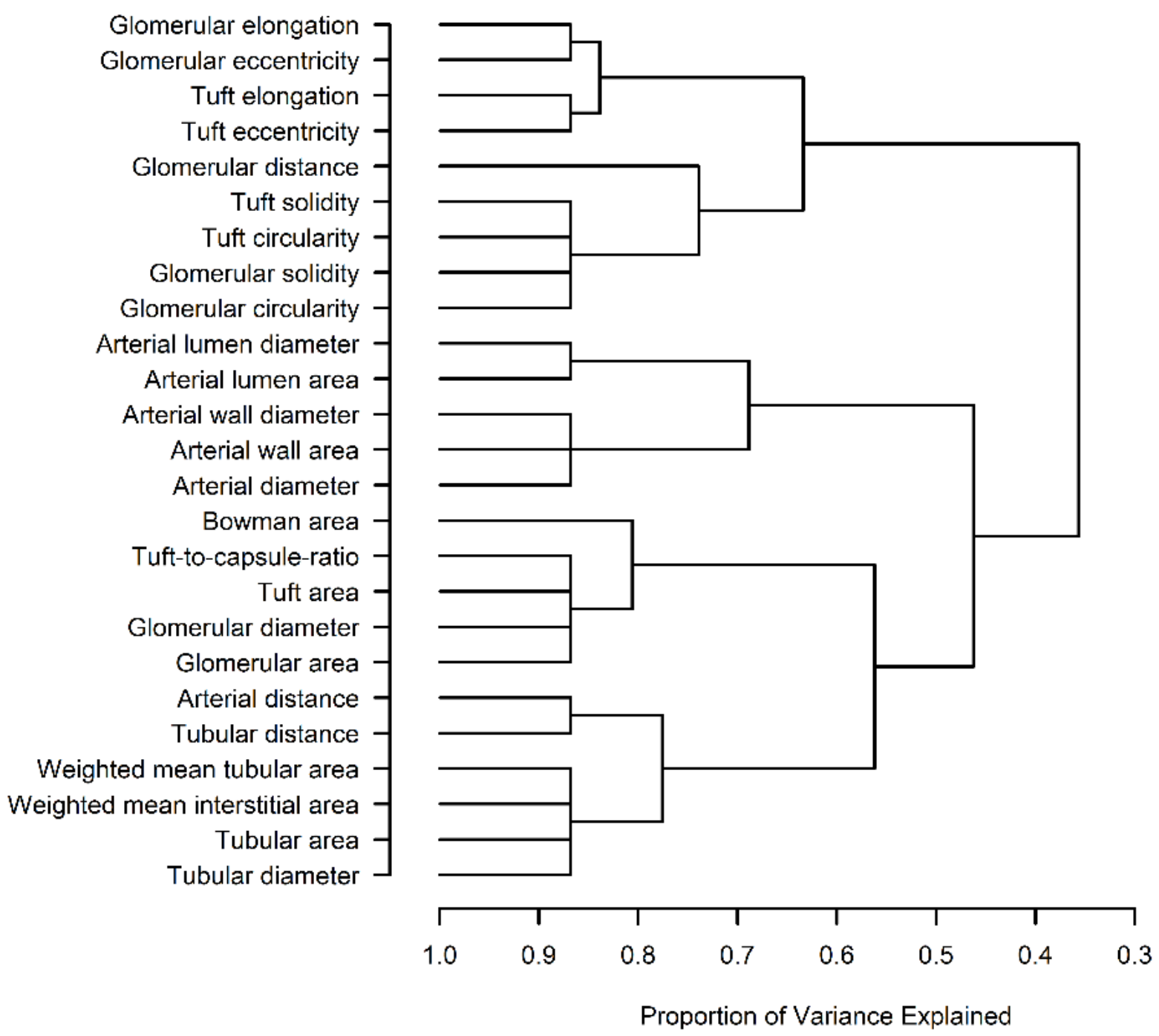

Supplementary Figure S3. Associations between pathomics variables, estimated glomerular function (eGFR, A) and proteinuria (B) at time of kidney biopsy.

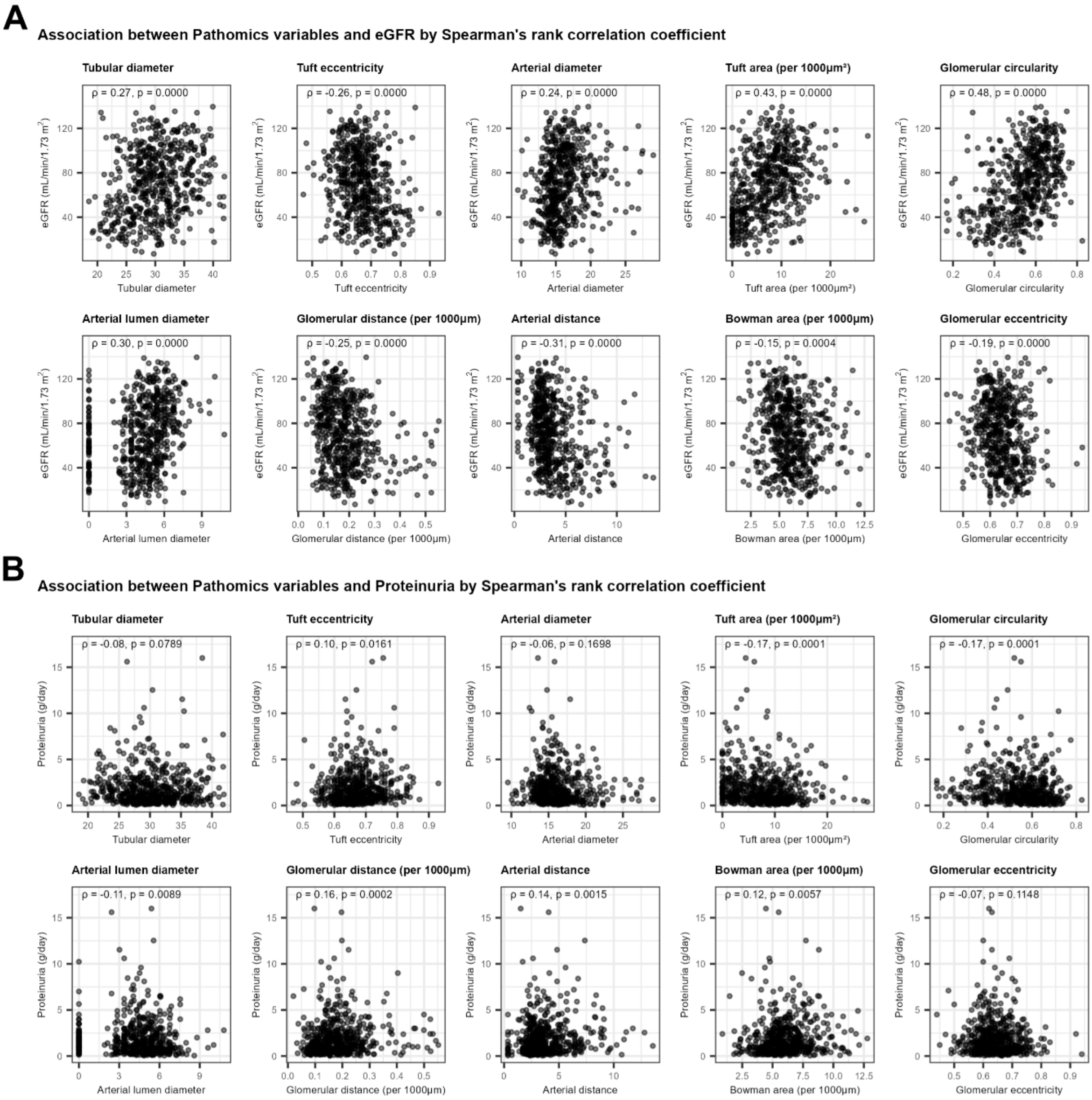

Supplement: Supplementary file 1 [file mmc1.pdf]
